# Impact of integrated clinical case exposure on phase I MBBS students in their learning process in biochemistry

**DOI:** 10.1186/s12909-025-08050-5

**Published:** 2025-11-06

**Authors:** Sushma BJ, Gaurav Mishra, RR Sukul, Sumit Parashar

**Affiliations:** 1 Department of Biochemistry, Pacific Medical College and Hospital, Udaipur, India; 2 Department of Radiodiagnosis, Jawaharlal Nehru Medical College, Datta Meghe Institute of Higher Education and Research, Wardha, India; 3 Department of Ophthalmology, Prasad Institute of Medical Sciences, Lucknow, India

**Keywords:** Integrated clinical case exposure, Clinical biochemistry, Didactic lectures, Phase i MBBS students, Pre-test and post-test scores

## Abstract

**Background:**

The integrated clinical case approach connects foundational medical concepts to real-life clinical scenarios, encouraging active problem-solving and interdisciplinary thinking. In contrast, traditional lecture-based teaching often presents subjects in isolation, without emphasizing clinical relevance. This study explores the potential impact of Early Clinical Exposure (ECE) through integrated clinical cases on biochemistry learning among Phase I MBBS students.

**Materials and methods background:**

The integrated clinical case approach connects foundational medical concepts to real-life clinical scenarios, encouraging active problem-solving and interdisciplinary thinking. In contrast, traditional: After obtaining informed consent, 100 Phase I MBBS students were randomly allocated into two groups: an ECE group and a non-ECE (traditional lecture-based) group, using a computer-generated random number table to minimize allocation bias. Both groups underwent pre-test and post-test assessments comprising multiple-choice questions (MCQs) (15 marks) and problem-based learning (PBL) questions (10 marks) designed to assess knowledge retention and application. A validated perception questionnaire was also administered post-intervention to gather students’ self-reported feedback on the learning experience.

**Results:**

Both groups demonstrated statistically significant improvement in post-test scores compared to pre-test scores (*p* < 0.001). The ECE group achieved higher mean post-test scores in MCQ (9.34 ± 1.45) and PBL (6.89 ± 0.99) compared to the non-ECE group (MCQ: 7.24 ± 1.29; PBL: 5.72 ± 0.73). An ANCOVA controlling for pre-test scores confirmed that these between-group differences in post-test scores were statistically significant for both MCQs (F(1,97) = 55.2, *p* < 0.001) and PBL scores (F(1,97) = 38.1, *p* < 0.001), indicating a significant effect of the intervention. Responses from the perception questionnaire indicated that most students felt ECE improved their understanding of biochemistry’s clinical relevance and enhanced motivation. Between 94% and 98% of students agreed that ECE helped them connect biochemistry concepts with clinical cases and aided content retention. However, these outcomes were subjective perceptions, not objectively measured indicators of engagement or motivation.

**Conclusion:**

The findings indicate that ECE was associated with improved knowledge retention and application of biochemistry concepts in clinical contexts compared to traditional lecture-based learning. While students reported increased motivation and appreciation of clinical relevance, these perceptions were self-reported and should be interpreted cautiously. Objective, long-term measures of engagement, clinical reasoning, and performance are recommended in future studies to substantiate these observations.

**Supplementary Information:**

The online version contains supplementary material available at 10.1186/s12909-025-08050-5.

## Introduction

Traditional didactic lectures, which form the cornerstone of medical education, often struggle to connect theoretical concepts with their practical clinical applications. This disconnect is particularly pronounced in the study of clinical biochemistry, a fundamental subject that plays a pivotal role in diagnosing and managing various diseases [1]. For Phase 1 MBBS students, the challenge lies in translating biochemical knowledge into real-world clinical practice, making the learning experience seem abstract and detached from patient care [2]. Early Clinical Exposure (ECE) has emerged as a promising educational strategy to address this issue. ECE involves exposing students to clinical environments early in their training, allowing them to observe and engage in patient care activities. This hands-on exposure enables students to better understand the relevance of basic sciences like biochemistry to clinical practice, thereby enhancing both learning and retention [3, 4].While the concept of ECE has been widely discussed in the literature, with several studies highlighting its benefits—such as improved clinical reasoning, better problem-solving skills, and increased motivation to learn [5, 6]—few have specifically focused on its impact on the comprehension and retention of clinical biochemistry concepts, particularly in the early years of medical education. Moreover, although some researchers have noted that ECE may be overwhelming and labour-intensive [7], these studies have primarily concentrated on a limited number of clinical disorders and have not thoroughly explored how ECE influences the integration of basic sciences into clinical practice across the full spectrum of clinical conditions. What sets this study apart from previous research is its specific focus on how early clinical exposure influences Phase 1 MBBS students’ understanding of clinical biochemistry—a subject that is foundational to diagnosing and managing diseases but is often perceived as detached from patient care. While many studies have advocated for the incorporation of clinical cases early in the curriculum, few have examined how ECE affects the retention and application of biochemistry concepts in real-world clinical contexts. This paper aims to fill this gap by investigating the direct impact of ECE on the comprehension, retention, and practical application of clinical biochemistry concepts among Phase 1 MBBS students. By comparing the outcomes of students exposed to clinical scenarios early in their education with those who received traditional didactic instruction, we seek to demonstrate the value of ECE in improving student engagement, knowledge retention, and the ability to apply theoretical concepts in clinical settings. Furthermore, this study also contributes to the literature by examining students’ feedback on ECE through a validated questionnaire based on a Likert scale. While the efficacy of ECE has been explored in broader terms, this research adds a specific dimension by capturing students’ perceptions and experiences of ECE, providing valuable insights into how this educational strategy is perceived in relation to the retention of biochemistry knowledge. Ultimately, this research underscores the importance of incorporating ECE into the medical curriculum to produce well-rounded, clinically proficient healthcare professionals. By addressing this gap, this study offers novel evidence on the role of early clinical exposure in enhancing the teaching and learning of clinical biochemistry, supporting the case for its broader integration into medical education.

## Materials and methods

This quasi-experimental study was conducted among 100 Phase I MBBS students (2021–22 batch) at Prasad Institute of Medical Sciences, Lucknow. Ethical approval was obtained from the Institutional Ethics Committee (Approval No.: PIMS/PO/572/2022, dated 16.03.2022), and the study adhered to institutional and national ethical guidelines.

### Study design and randomization

Participants were randomly allocated into two equal groups: the Early Clinical Exposure (ECE) group and the non-ECE (traditional lecture-based) group. Randomization was carried out using a computer-generated random number table by an independent faculty member not involved in the intervention or assessment to ensure allocation concealment. Students from other batches or those absent during the intervention were excluded.

#### Assessment tools

Student performance was evaluated using two validated tools:

1. Multiple Choice Questions (MCQs): Fifteen MCQs (1 mark each) were designed to assess recognition and application-level cognitive skills. Questions were reviewed and validated by a panel of senior faculty from Biochemistry and Internal Medicine for content relevance, clinical alignment, and appropriate difficulty level.
2. Problem-Based Learning (PBL) Questions: Two clinical vignette-based scenarios (5 marks each) were used to assess the ability to integrate biochemical knowledge into clinical reasoning.
3. The same assessment format was used at three time points:

- Pre-test (baseline, before Session 1).
- Post-test 1 (after Session 1).
- Post-test 2 (after Session 2, following crossover of groups).

Baseline equivalence between groups was confirmed using an independent t-test on pre-test scores, showing no significant difference.

#### Intervention procedure

Each instructional session lasted two hours per week.

- The non-ECE group attended traditional didactic lectures.
- The ECE group participated in integrated, clinical case–based learning sessions.

After Session 1, the groups were crossed over, and Session 2 followed the same teaching format and assessment structure.

#### ECE session design

ECE modules were jointly developed by faculty from Biochemistry and Internal Medicine through focused group discussions. For active participation, the ECE group was divided into five subgroups of ten students each.

1. Session 1: Jaundice (Competency BI11.7) — Learning objectives included understanding the biochemical basis of jaundice, interpreting liver function tests, and correlating biochemical findings with clinical features. Real patient cases (e.g., neonatal or hemolytic jaundice) were used, supported by discussions on bilirubin metabolism, mock clinical examinations, lab interpretation, differential diagnosis, and management strategies.
2. Session 2: Diabetes Mellitus (Competency BI3.10) — Learning objectives included understanding glucose metabolism, interpreting blood glucose and HbA1c results, and applying biochemical principles to clinical diagnosis and management. The session followed the same structure as Session 1, using real patient cases (e.g., type 2 diabetes mellitus).

Each ECE session concluded with a structured summary and student reflective writing.

### Student feedback and questionnaire validation

At the end of both ECE sessions, student feedback was collected via a 9-item questionnaire on a 5-point Likert scale (1 = Strongly Disagree to 5 = Strongly Agree). The questionnaire was validated by experts from the Medical Education Unit and relevant departments, and its reliability was confirmed by a test–retest method (Cronbach’s alpha = 0.85), indicating high internal consistency.

### Statistical analysis

Data were analysed using SPSS v25.0. Paired t-tests were applied for within-group comparisons (pre- vs. post-tests), and independent t-tests for between-group comparisons. A *p*-value < 0.05 was considered statistically significant.

**Fig. 1:**
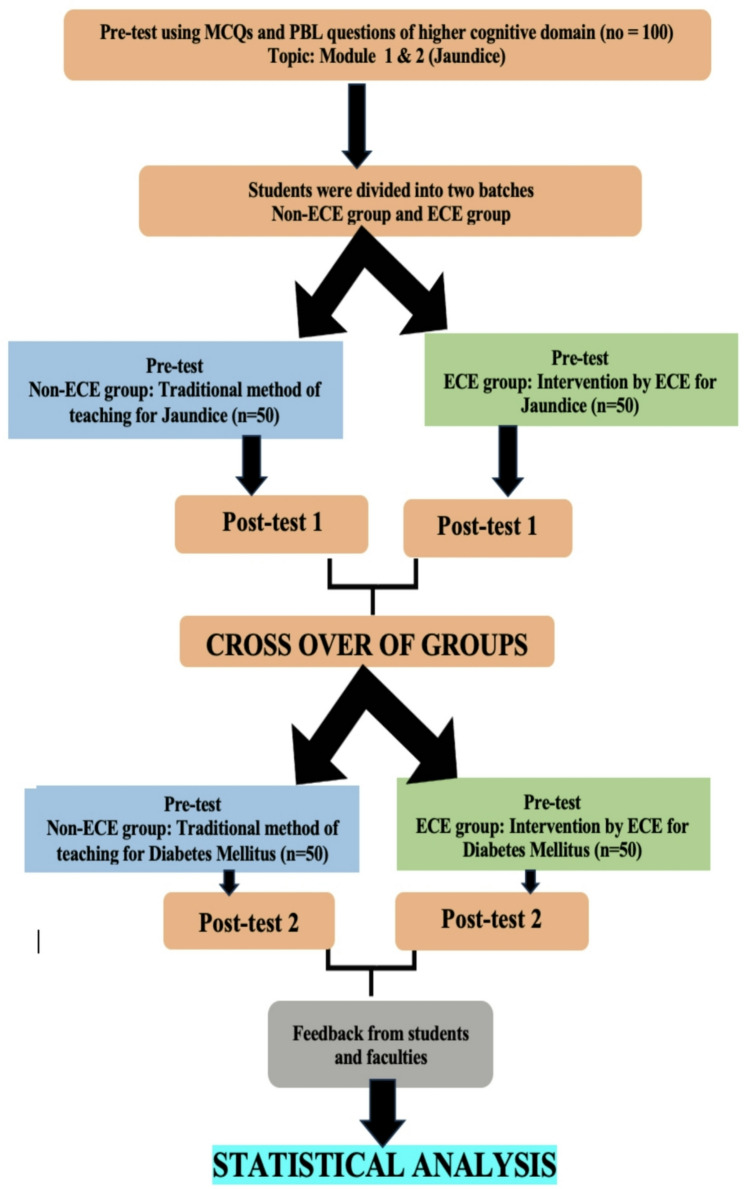
Flow Chart representing the Study Methodology

## Results

A total of 100 Phase I MBBS (2021–22 batch) students participated in the study after providing voluntary consent. At baseline, all students completed a pre-test comprising MCQs and case study–based (PBL-style) questions. The normality of the score distributions was confirmed using the Shapiro-Wilk test (*p* > 0.05 for all groups), validating the use of parametric tests.

Table 1 shows the comparison of pre-test and post-test scores for Session 1 (Topic: Jaundice). Both groups demonstrated statistically significant improvement in post-test scores compared to pre-test scores (*p* < 0.001). To directly compare the effectiveness of the two teaching methods, an Analysis of Covariance (ANCOVA) was conducted with the post-test score as the dependent variable and the pre-test score as the covariate. This analysis confirmed that the ECE group’s post-test scores were significantly higher than the non-ECE group’s for both MCQs (F(1,97) = 55.2, *p* < 0.001) and Case Studies (F(1,97) = 38.1, *p* < 0.001) after controlling for baseline knowledge.

**Table 1:** Comparison of pre-test and post-test scores of session 1 between Non-ECE and ECE group (topic: Jaundice)

Assessment Method	Non ECE Group		ECE Group	
	Pre-test (*n* = 50)	Post test (*n* = 50)	Pre-test (*n* = 50)	Post test (*n* = 50)
MCQ (15 marks)	2.74 ± 0.93	7.24 ± 1.28**	2.82 ± 0.96	9.34 ± 1.45**
Case Study (10 marks)	0.88 ± 0.84	5.72 ± 0.72**	0.98 ± 0.80	6.89 ± 0.98**

**p value < 0.001, Highly Significant (within-group comparison via paired t-test)

Table 2 presents the results for Session 2 (Topic: Diabetes Mellitus) after crossover. Again, both groups showed statistically significant improvement from pre-test to post-test (*p* < 0.001). An identical ANCOVA model was applied to the Session 2 data. The results again showed a statistically significant effect of the intervention, with the ECE group outperforming the non-ECE group on both the MCQ (F(1,97) = 25.8, *p* < 0.001) and Case Study (F(1,97) = 20.4, *p* < 0.001) post-tests after adjusting for pre-test scores.

**Table 2:** Comparison of pre-test and post-test scores of session 2 between Non-ECE and ECE group (topic: diabetes mellitus)

Assessment method	Non ECE group		ECE group	
	Pre-test (*n* = 50)	Post test (*n* = 50)	Pre-test (*n* = 50)	Post test (*n* = 50)
MCQ (15 marks)	2.16 ± 1.18	8.72 ± 0.85**	2.28 ± 0.85	10.0 ± 1.88**
Case Study (10 marks)	0.96 ± 0.63	5.98 ± 0.98**	0.96 ± 0.69	7.02 ± 1.30**

**p value < 0.001, HS (within-group comparison via paired t-test)

Table 3 summarises student perceptions towards ECE. Responses to the validated 9-item questionnaire indicated overwhelmingly positive feedback. For example, 100% of students strongly agreed that ECE helped them understand the importance of biochemistry and preferred it over traditional lectures. Between 94% and 98% reported that ECE improved their attention span, retention of topics, and ability to correlate biochemistry with clinical cases and laboratory results. While these findings suggest that ECE may contribute towards enhancing engagement, retention, and application of concepts, it is important to note that these perceptions are self-reported and were not objectively measured.

**Table 3:** Shows students perceptions towards Early Clinical Exposure

Sl. No	Question	Strongly Agree	Agree	Neutral	Strongly Disagree	Disagree
1	Did ECE helped to understand the importance of Biochemistry	100%	0	0	0	0
2	Did ECE increased attention span in class	94%	6%	0	0	0
3	Did ECE helped to improve interaction with teacher	100%	0	0	0	0
4	Did ECE helped in retention of topic better	98%	2%	0	0	0
5	The time allocated for ECE was adequate	89%	7%	2%	0	0
6	Do you prefer ECE over didactic lecture i.e biochemistry lectures without ECE	100%	0	0	0	0
7	ECE helped me to correlate biochemistry with clinical case	98%	2%	0	0	0
8	Do you think ECE was helpful to correlate the laboratory results with the clinical case	96%	2%	2%	0	0
9	Do you think learning biochemistry will help you to manage the patients in front line	94%	2%	4%	0	0

## Discussion

The present study explored the potential impact of Early Clinical Exposure (ECE) on Phase I MBBS students’ learning outcomes in biochemistry. Statistically significant improvements were observed in post-test scores for both groups. Critically, the ANCOVA analysis, which controls for baseline differences, provides robust evidence that the ECE intervention was associated with significantly greater improvement in scores compared to traditional lectures. The relatively higher post-test scores in the ECE group suggest that ECE may contribute to better short-term knowledge retention and the ability to apply biochemical concepts to clinical contexts.

These findings are in line with existing literature demonstrating that integrating clinical experiences with pre-clinical education can improve students’ understanding and retention of medical knowledge [3, 4]. A number of studies have highlighted the effectiveness of ECE in bridging the gap between theoretical knowledge and clinical practice. For instance, Dhonde et al. (2015) explored the role of ECE in biochemistry and other pre-clinical subjects and found that students who participated in clinical exposure showed better integration of theoretical knowledge in clinical settings [8–10]. This supports our observation that ECE may help improve not only knowledge retention but also the ability to apply this knowledge to clinical cases. Similarly, Shivkar et al. (2020) demonstrated that ECE in biochemistry increased student engagement by fostering connections between theoretical content and its clinical applications, resulting in improved retention of biochemistry concepts [11]. Their study highlighted how ECE facilitated a deeper understanding of biochemistry, aligning with our finding that students in the ECE group scored relatively higher on both MCQ and case study assessments.

In addition, Gupta et al. (2020) noted that ECE, by making content more interactive and relatable, increased students’ enthusiasm and attention in the classroom, which was particularly beneficial in biochemistry, a subject often considered abstract and disconnected from clinical practice [12]. In our study, many students reported perceived benefits such as increased attention span (94%) and improved interaction with the teacher (100%) during ECE sessions. However, it is important to note that these outcomes were derived from self-reported perception questionnaires rather than objective measures, and therefore should be interpreted cautiously.

To support transparency and reproducibility, the pre- and post-test questions (MCQs and PBL items) used in this study will be provided as supplementary material. These items were designed to evaluate two primary learning domains: (a) Knowledge retention – assessed through MCQs targeting recall and recognition of key biochemical concepts relevant to jaundice and diabetes and (b) Application of knowledge – assessed through case-based PBL questions requiring interpretation of clinical data and integration of biochemical principles into clinical reasoning.

Similarly, anonymized ECE case materials for Jaundice and Diabetes Mellitus (adapted from real patient scenarios) will be made available as supplementary resources to support other educators in developing clinically integrated sessions.

In the context of previous research, several studies have demonstrated benefits of ECE in pre-clinical teaching [5, 6, 13–18]. Our study adds to the existing literature by employing a crossover design where each student experienced both teaching formats, reducing variability from baseline differences between groups. Additionally, by incorporating both MCQ and PBL assessments and a robust ANCOVA analysis, we provide stronger evidence of the intervention’s effect on both factual recall and applied reasoning, which are less frequently reported in earlier ECE studies in biochemistry.

In summary, while our results provide statistical evidence that ECE may contribute to improved short-term knowledge retention and application in biochemistry, conclusions about motivation, engagement, and clinical relevance are based on subjective perceptions and should be interpreted with caution. The supplementary materials provided may serve as a resource for biochemistry educators seeking to implement ECE in their teaching.

## Conclusion

In conclusion, this study provides evidence that Early Clinical Exposure (ECE) was associated with significantly greater short-term gains in biochemistry knowledge scores compared to traditional didactic methods. ECE was also perceived by students to increase engagement and aid in better retention and application of clinical knowledge, making it a promising educational strategy. Given these favourable results, medical educators and policymakers should consider incorporating ECE into the curriculum to bridge the gap between theoretical knowledge and clinical practice. However, the long-term impact on clinical reasoning and performance remains to be established.

### Limitations of our study

1. Small Sample Size: The limited number of participants may not fully capture the diversity of student learning abilities and prior knowledge. This restricts the generalizability of the findings.
2. Short Study Duration: The brief duration of the study did not allow for assessment of long-term knowledge retention or evaluation of the sustained impact of early clinical exposure (ECE) on clinical performance.
3. Lack of Exploration of Teaching Variations: The study did not examine how different teaching methods within the ECE framework might influence student outcomes, particularly for those with varied learning preferences.
4. Faculty Training Not Assessed: The influence of faculty training and preparedness on the effectiveness and consistency of ECE delivery was not evaluated, which may affect the quality of the student experience.
5. Limited Insight into Learning Processes: While student perceptions were collected, the deeper cognitive and experiential learning processes associated with ECE were not thoroughly explored. Qualitative interviews could provide richer insights in future studies.
6. Single-Institution Study: Conducted at one medical college, the findings may be influenced by local institutional and faculty factors, limiting their generalizability to other settings.

Future research should investigate whether the observed gains persist over time and whether early exposure to clinical cases translates into improved clinical reasoning and patient care skills in later years.

## Supplementary Information

Supplementary Material 1.

## Data Availability

Data was entered into Microsoft Excel and Google Sheet and is available from the corresponding author on reasonable request.
