# Supplementary figures and images for: Impact of integrated clinical case exposure on phase I MBBS students in their learning process in biochemistry
Source: BMC Med Educ. 2025 Nov 6;25:1557. doi: 10.1186/s12909-025-08050-5 (PMC12590858; doi:10.1186/s12909-025-08050-5)

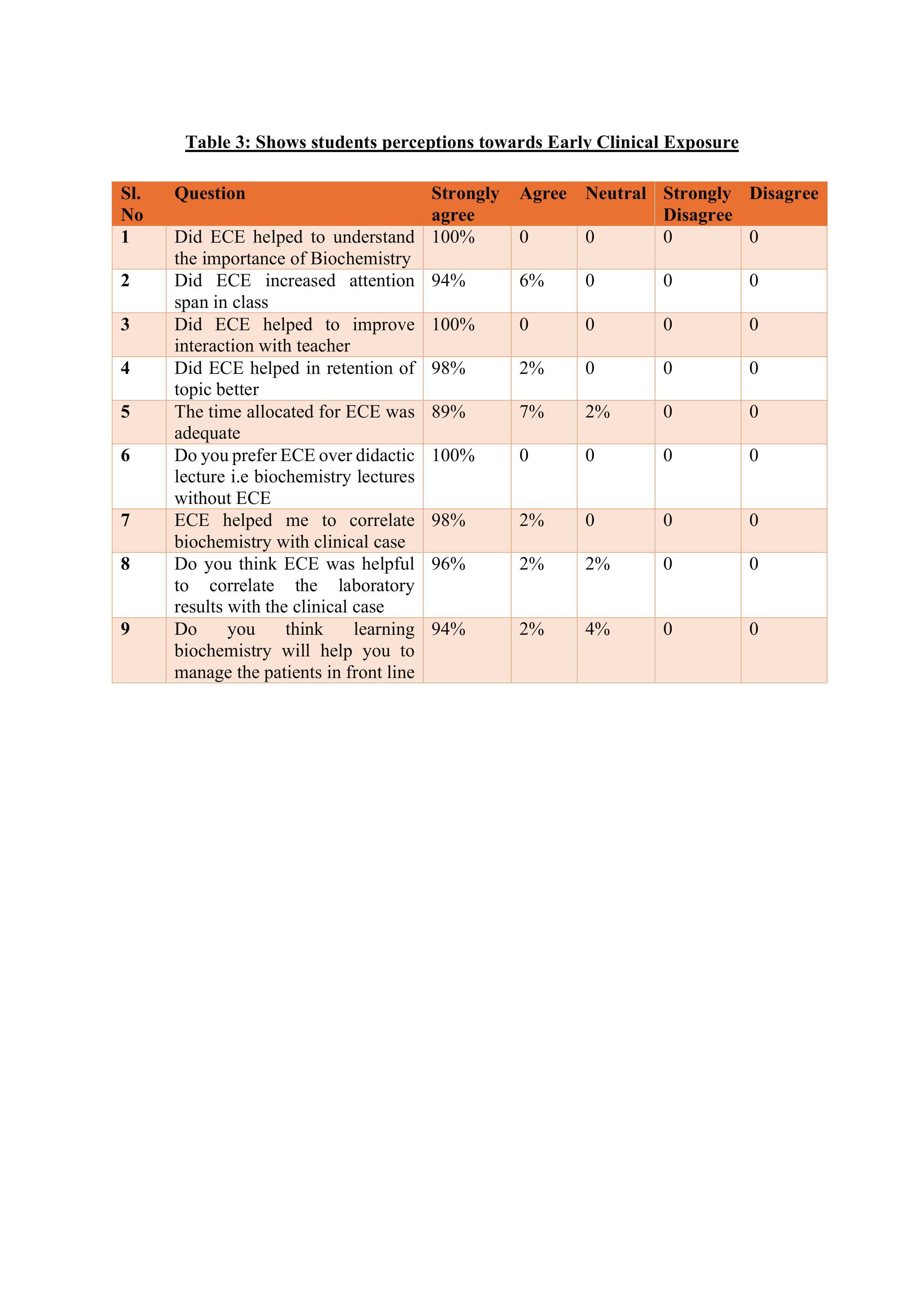

Supplement: Supplementary file 1 — Supplementary Material 1. [file 12909_2025_8050_MOESM1_ESM.jpeg]
